# Successful laparoscopic retroperitoneal tumor resection using mixed reality and guiding marker techniques

**DOI:** 10.1002/iju5.12735

**Published:** 2024-06-03

**Authors:** Yoichiro Tohi, Homare Okazoe, Katsuya Mitamura, Yu Osaki, Kenichi Tanaka, Yuki Matsuoka, Yoshihiro Nishiyama, Kenji Kanenishi, Mikio Sugimoto

**Affiliations:** ^1^ Department of Urology, Faculty of Medicine Kagawa University Kita‐gun Kagawa Japan; ^2^ Department of Radiology, Faculty of Medicine Kagawa University Kita‐gun Kagawa Japan; ^3^ Department of Perinatology and Gynecology, Faculty of Medicine Kagawa University Kita‐gun Kagawa Japan

**Keywords:** Holoeyes, mixed reality, retroperitoneal

## Abstract

**Introduction:**

Small tumors may be difficult to identify visually and require preoperative effort to locate. Recent advancements in mixed reality technology have improved surgical accuracy in various departments. Here, we present the application of mixed reality‐assisted surgery and a guiding marker in the case of small retroperitoneal metastasis of uterine cancer.

**Case presentation:**

A 67‐year‐old female with a history of uterine cancer had a retroperitoneal metastasis in the lateroconal fascia near the right diaphragm, measuring 2 cm and infiltrating the peritoneum. We performed precise surgical planning using the preoperative mixed reality software “Holoeyes” on a head‐mounted display called HoloLens2. Novel techniques, including ultrasonography‐guided placement of a guiding marker and strategic port‐site placement facilitated by HoloLens2, ensured accurate tumor identification and laparoscopic resection with minimal blood loss and no intraoperative complications.

**Conclusion:**

The use of mixed reality‐assisted surgery and a guiding marker effectively enhanced the precision of retroperitoneal tumor resection.

**Keynote message**

In the present case study of uterine cancer metastasis, the use of mixed reality (MR) technology and a guiding marker significantly enhanced the precision of localization of small retroperitoneal tumors. This approach augments the accuracy of tumor identification and ensures patient safety by minimizing unnecessary exposure.

**Abbreviations & Acronyms**
3D: three‐dimensional
CT: computed tomography
HMD: head‐mounted display
MR: mixed reality
STL: Standard Triangulated Language

## Introduction

Small tumors may be difficult to identify visually and require intraoperative efforts to identify.^1^ Retroperitoneal tumors are particularly difficult to identify because they are surrounded by fat and because the retroperitoneal space has few surgical landmarks. Unnecessary exposure owing to misidentification of the tumor site should be avoided in patients with retroperitoneal tumors. Therefore, confirming the precise surgical level using radiographic examinations is essential. Research using various MR technologies has recently advanced in the medical field, including urology,^2^, ^3^, ^4^, ^5^ and urological surgeons perceive MR as a useful and interesting tool in the preoperative setting in partial nephrectomy.^6^ Therefore, we aimed to explore the application of MR in the surgical management of retroperitoneal tumors. Herein, we present a case in which MR‐assisted surgery for retroperitoneal metastasis of uterine cancer was successful.

## Case presentation

A 67‐year‐old female with retroperitoneal metastasis of uterine cancer was referred from our gynecology department for tumor resection. The patient had a history of surgery for uterine cancer with a scar from a midline lower abdominal incision. The patient's height, weight, and body mass index were 150.9 cm, 58.3 kg, and 25.6 kg/m^2^, respectively.

**Fig. 1:**
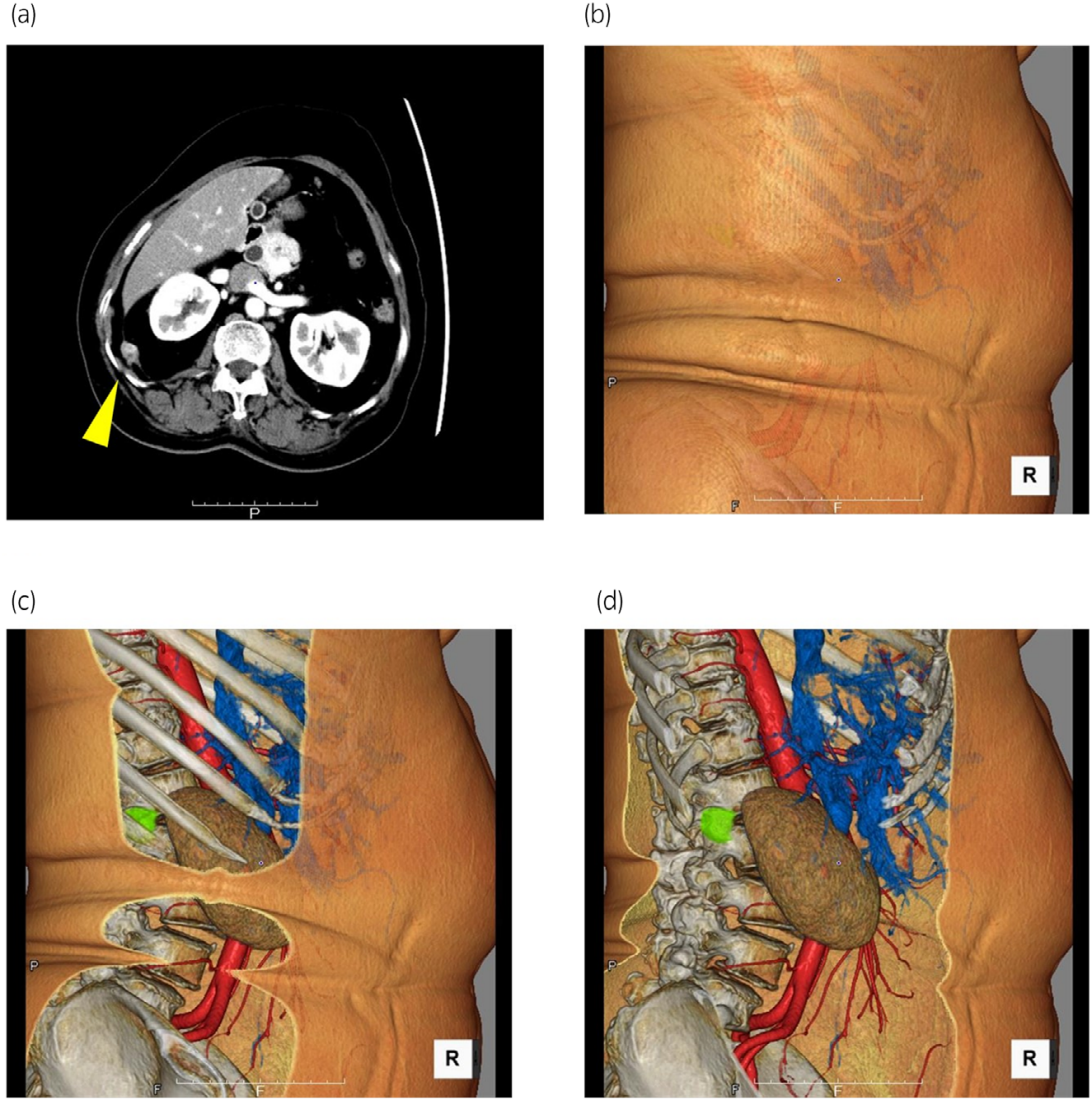
(a) A contrast‐enhanced CT scan shows retroperitoneal metastasis in the lateroconal fascia close to the right diaphragm, partly infiltrating the peritoneum. The tumor measured 2 cm in size and showed contrast enhancement. (b–d) STL data (3D data) were produced using the workstation. (b–d) 3D data from the skin to inside the body (the green sphere indicates the tumor).

A contrast‐enhanced CT scan revealed retroperitoneal metastasis in the lateroconal fascia close to the right diaphragm, partly infiltrating the peritoneum. The tumor measured 2.0 × 1.9 × 1.5 cm in size and showed contrast enhancement (Fig. 1a). Because the tumor was located in the lateroconal fascia surrounded by fat on CT, it was expected to be difficult to identify.

**Fig. 2:**
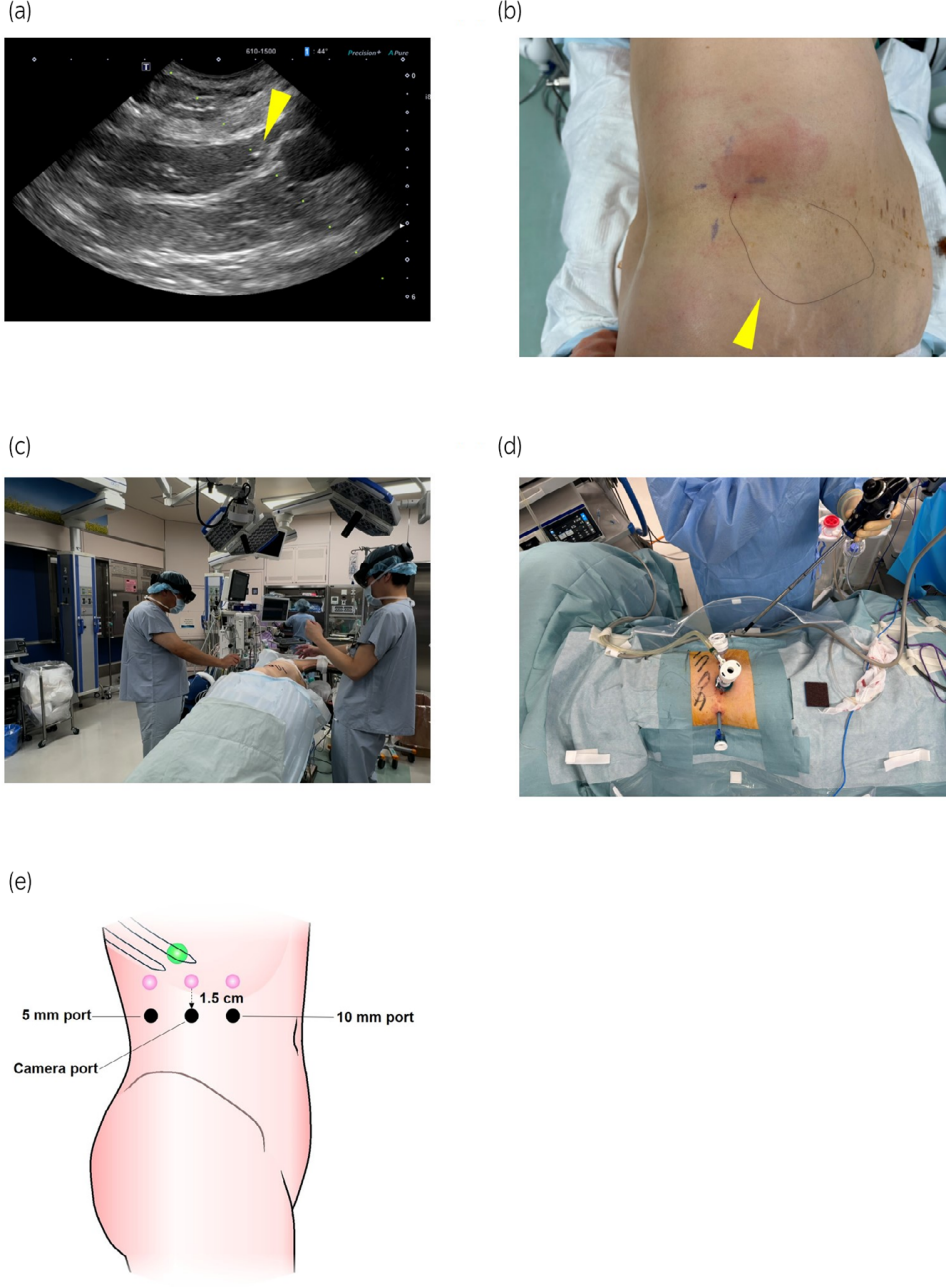
(a) A guiding marker was placed 1 cm caudal to the tumor within the flank pad under echo guidance (arrow indicates marker). (b) The guiding marker comprises a nylon thread and a stainless marker at the tip. The thread of the guiding marker was observed in the surgical field (arrow indicates marker). (c) The surgical team identified the port sites using the HoloLens2 HMD. (d) The placed ports. (e) The port was placed 1.5 cm caudal to our usual laparoscopic nephrectomy port (the pink spheres) (the green sphere indicates the tumor. The black sphere indicates the port position in the present case).

**Fig. 3:**
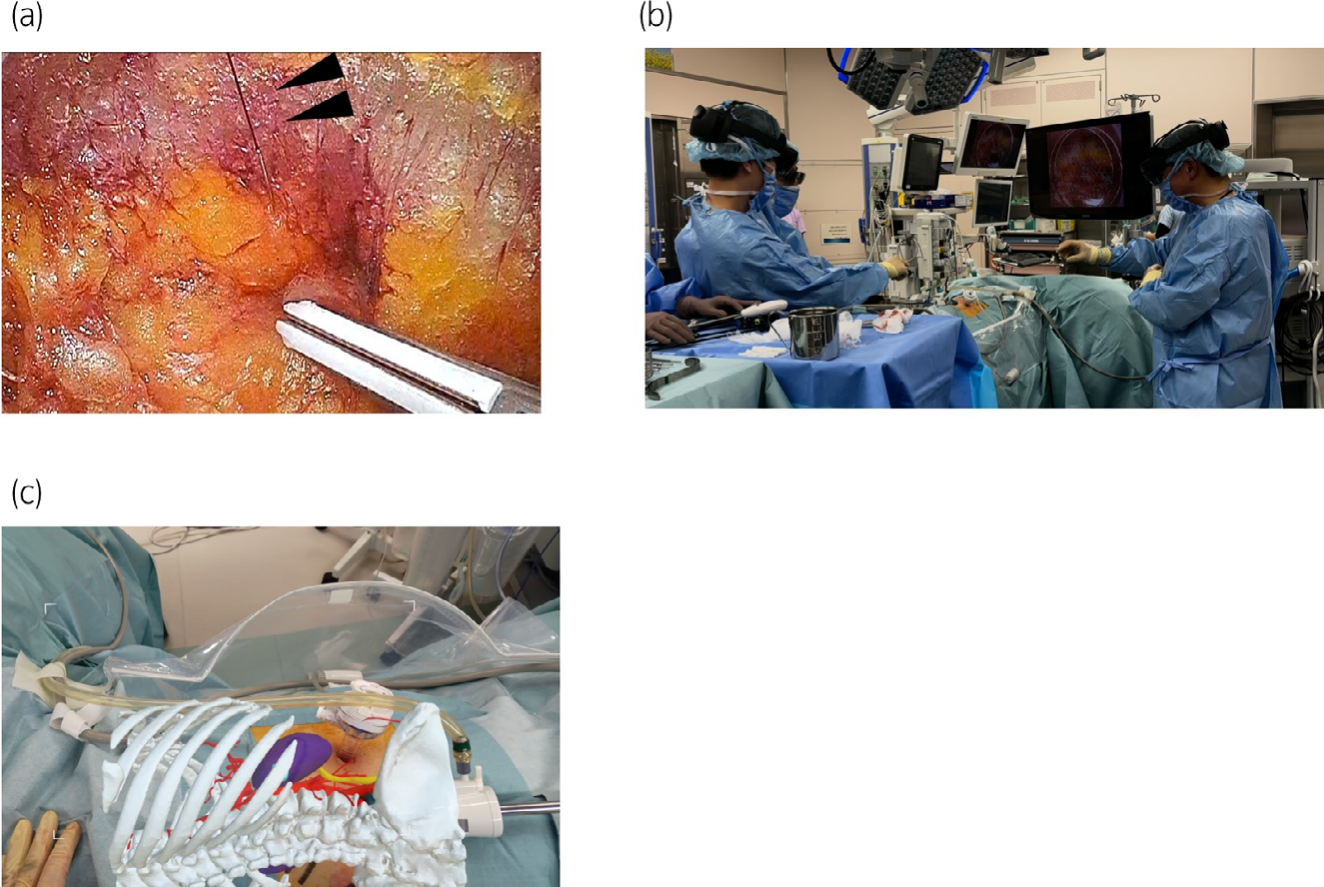
(a) The guiding marker placed on the flank pad within the surgical field was observed on a fiberscope (arrows indicate the thread of the guiding marker). (b) The surgical team used HoloLens2 to identify the location of the tumor again. (c) The retroperitoneal tumor was identified using the HoloLens2 HMD. The blue sphere behind the 11th rib indicates the tumor.

Preoperative 3D CT images were generated, and STL data (3D data) were produced using a workstation (Fig. 1b–d). The generated STL data were then imported into the augmented reality software “Holoeyes” (Holoeyes Inc., Tokyo, Japan) and deployed on a HMD called HoloLens2 (Microsoft Corporation, Redmond, WA, USA).^7^, ^8^ Preoperatively, the surgical team used HoloLens2 to identify the retroperitoneal tumor. Following anesthesia induction, a guiding marker (Guiding‐Marker SYSTEM, Hakko Co. Ltd., Nagano, Japan) (21G × 150 mm) was precisely placed 1 cm caudal to the tumor within the adipose tissue (flank pad) under ultrasonographic guidance (Fig. 2a,b). Subsequently, the port site was determined using HoloLens2, enabling the 3D observation of the CT data (Fig. 2c). This process revealed that the conventional port site located directly below the costal arch for laparoscopic nephrectomy was near the tumor. Therefore, the port site was set 1.5 cm caudal to this location (Fig. 2d). After placing the camera port, the retroperitoneum was briefly expanded using a pre‐peritoneal distension balloon. Subsequently, 5 and 12 mm ports for the left and right hands, respectively, were placed sequentially. The guiding marker placed on the flank pad within the surgical field was observed on a flexible endoscope (Fig. 3a). The flank pad was incised 1 cm caudal to the tip of the guiding marker to expose the lateroconal fascia, which was then incised. The surgical team used HoloLens2 to identify the location of the tumor again (Fig. 3b,c). The fat layer surrounding the tumor was dissected, and the peritoneum to which the tumor was adhered was partially excised. The tumor was removed en bloc along with the guiding marker and was then placed in an E·Z PURSE (Hakko Medical, Nagano, Japan) and extracted externally. The specimen was sliced to confirm the presence of cancer. The peritoneal defect was closed by continuous suturing using a monofilament thread. The laparoscopic operative time was 1 h and 39 min, with no intraoperative complications and minimal blood loss. No postoperative complications occurred. The pathological results were consistent with a diagnosis of uterine cancer metastasis. The resection margin was negative.

## Discussion

Herein, we report the use of MR to support the intraoperative localization of small retroperitoneal tumors. Based on the present case of retroperitoneal metastasis of uterine cancer, the integration of the MR technique and the placement of a guiding marker provide additional insights into the surgical management of retroperitoneal tumors. This new approach improves the accuracy of tumor localization and provides safety for the patient because it allows for a learner procedure. Working with the radiology department for preoperative preparation and extraction of 3D data requires time and effort because such a procedure is not observed in daily clinical practice. Manual alignment can introduce tracking errors of a few millimeters.^9^ To minimize tracking errors, we implemented three additional ideas. First, conducting preoperative CT in the same position where surgery was performed, specifically the left lateral decubitus position in our case, minimized localization errors, further demonstrating the importance of careful planning. Second, the difficulty in overlaying MR's 3D imaging with the patient's anatomy owing to the absence of landmarks on the patient's body was addressed by marking the ribs on the patient, thus facilitating calibration with the MR images. These procedures were crucial in determining optimal port positions and ensuring that the surgical team proceeded with confidence in the accuracy of tumor localization. Third, a guiding marker was placed preoperatively. The use of guiding markers, which identify the location of lung tumors,^1^ was adopted in the present case to identify the location of the retroperitoneal tumor. The decision to place the guiding marker under ultrasonographic guidance after anesthesia induction was based on its ability to mitigate the risks of migration and positional changes, ensuring the effectiveness of the marker in guiding the surgical approach. This strategic placement, performed immediately before surgery, underscores the value of real‐time intraoperative guidance. The tumor was confirmed by ultrasonography in the present case; however, in cases where this is impossible, MR can be used for the tumor identification. Furthermore, in the present case, MR confirmed that the mass observed by ultrasonography was the target tumor in CT. Studies on MR are significantly increasing; however, there is a lack of clinical research demonstrating its benefits.^10^ Therefore, the findings of the present study provide valuable insights into surgical approaches in the field of urology.

Notably, the need for specialized equipment and additional procedures beyond those observed in daily clinical practice may limit the broad applicability of our findings. However, the enhanced accuracy of tumor localization could lead to improved patient safety and outcomes.

In conclusion, we present the use of MR and a guiding marker in addressing retroperitoneal tumors, highlighting a novel approach for improving surgical precision and reducing the risk of unnecessary exposure by enhancing tumor localization and surgical planning.

## Author contributions

Yoichiro Tohi: Conceptualization; writing – original draft. Homare Okazoe: Conceptualization; writing – review and editing. Katsuya Mitamura: Writing – review and editing. Yu Osaki: Writing – review and editing. Kenichi Tanaka: Writing – review and editing. Yuki Matsuoka: Writing – review and editing. Yoshihiro Nishiyama: Writing – review and editing. Kenji Kanenishi: Writing – review and editing. Mikio Sugimoto: Supervision.

## Conflict of interest

Mikio Sugimoto is an Editorial Board member of International Journal of Urology and a co‐author of this article. To minimize bias, they were excluded from all editorial decision‐making related to the acceptance of this article for publication.

## Approval of the research protocol by an Institutional Reviewer Board

Not applicable.

## Informed consent

Informed consent was obtained from the patient for the publication of this case report and the accompanying images.

## Registry and the Registration No. of the study/trial

Not applicable.
